# Sexual Dimorphism of the Heart: Genetics, Epigenetics, and Development

**DOI:** 10.3389/fcvm.2021.668252

**Published:** 2021-05-26

**Authors:** Daniel F. Deegan, Priya Nigam, Nora Engel

**Affiliations:** Lewis Katz School of Medicine, Fels Institute for Cancer Research, Temple University, Philadelphia, PA, United States

**Keywords:** sex differences, cardiac, sex-biased expression, embryogenesis, developmental origin of disease, epigenetics

## Abstract

The democratization of genomic technologies has revealed profound sex biases in expression patterns in every adult tissue, even in organs with no conspicuous differences, such as the heart. With the increasing awareness of the disparities in cardiac pathophysiology between males and females, there are exciting opportunities to explore how sex differences in the heart are established developmentally. Although sexual dimorphism is traditionally attributed to hormonal influence, expression and epigenetic sex biases observed in early cardiac development can only be accounted for by the difference in sex chromosome composition, i.e., XX in females and XY in males. In fact, genes linked to the X and Y chromosomes, many of which encode regulatory factors, are expressed in cardiac progenitor cells and at every subsequent developmental stage. The effect of the sex chromosome composition may explain why many congenital heart defects originating before gonad formation exhibit sex biases in presentation, mortality, and morbidity. Some transcriptional and epigenetic sex biases established soon after fertilization persist in cardiac lineages, suggesting that early epigenetic events are perpetuated beyond early embryogenesis. Importantly, when sex hormones begin to circulate, they encounter a cardiac genome that is already functionally distinct between the sexes. Although there is a wealth of knowledge on the effects of sex hormones on cardiac function, we propose that sex chromosome-linked genes and their downstream targets also contribute to the differences between male and female hearts. Moreover, identifying how hormones influence sex chromosome effects, whether antagonistically or synergistically, will enhance our understanding of how sex disparities are established. We also explore the possibility that sexual dimorphism of the developing heart predicts sex-specific responses to environmental signals and foreshadows sex-biased health-related outcomes after birth.

## Introduction

**Figure 1:**
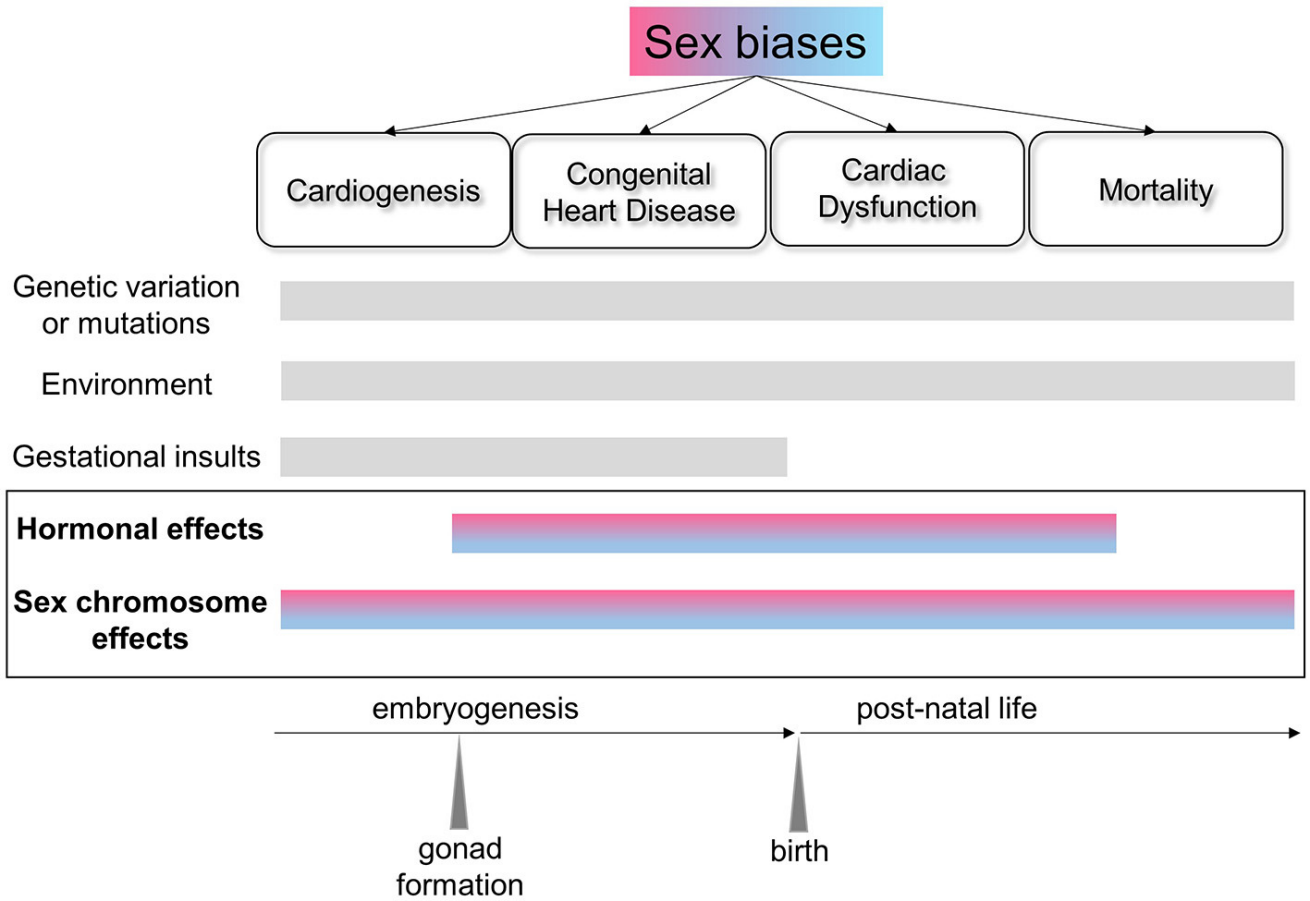
Schematic of the factors affecting sex biases in the heart across the lifespan. Sexual dimorphism exists at the molecular level from early cardiac development and across the lifespan, and is reflected in the sex biases evident in congenital heart disorders and cardiovascular health and disease. In addition to genetic, environmental and gestational factors, sex chromosome-linked genes and their interactions with hormonal effects are critical in establishing sex differences in the heart.

Biological sex has long been known to affect the epidemiology, clinical manifestation, pathophysiology, and response to treatment for cardiovascular disorders (1), yet the basic mechanisms underlying these differences remain unknown. Filling this knowledge gap and elucidating sex-biased protective factors should lead to better, more selective treatments for both sexes. Research contemplating sex differences has only recently become somewhat more mainstream, with the mandate from federal agencies to consider sex as a vital biological factor (2). Often, however, studies have included both sexes without sufficient power to perform meaningful comparisons (3). Sex chromosomes are often excluded from genomic analyses and association studies, limiting the ability to identify sex-biased risk factors. Furthermore, many cardiovascular diseases are polygenic and multifactorial, posing additional challenges for understanding the mechanisms leading to sex disparities (Figure 1).

## Sex Biases in Healthy Hearts

There are baseline sex differences in healthy adults in cardiac structure and function, as shown in both humans and rodents (4). Many cellular processes, such as rhythmicity, lipid metabolism, regenerative capacity and fibrosis, differ between male and female cardiac cells (5–7). Moreover, recent studies have shown a surprising amount of transcriptional and epigenomic variability between male and female hearts, emanating from both the sex chromosomes and the autosomes (8–15). Efforts to connect the functional differences between males and females to the transcriptional biases are ongoing, although determining whether those biases are causal will require more extensive studies.

Traditionally, sex differences in cardiac function are solely attributed to the influence of sex hormones. In fact, a wealth of information has accrued over many decades attesting to the effects of androgen and estrogen on cardiomyocytes in humans and model organisms (16–18). These studies have elucidated the genomic effects of sex steroids mediated by their respective receptors, as well as non-genomic activity. However, some of the transcriptional sex biases are independent of hormones (19, 20). A study on sex biases from the Genotype-Tissue Expression (GTEx) Project found that only one-third of differentially expressed autosomal genes across all human somatic tissues contain estrogen or androgen response elements, suggesting either indirect hormonal influence or regulation by other factors altogether (14).

Even the demonstrably powerful effects of sex steroids on the heart are not completely understood. Both males and females produce estrogens and androgens, albeit at different levels, but their effects are rarely studied in both sexes. In one exception to this trend, estrogen was shown to have opposite effects on intracellular signaling in male and female mouse cardiac myocytes, even though the levels of estrogen receptors do not differ (20, 21). Therefore, the differences in the responses to estrogen must be related to the sex-specific hormonal levels, but this begs the question of how cardiac cells sense hormonal dosages. Are there uncharacterized receptor variants, conformations or post-translational modifications that are sex-biased? Do the higher levels of estrogen in females, for example, activate a wider range of genes? Are the genomes in male and female cardiac cells epigenetically primed to activate different target genes in response to hormones? Recent studies suggest that cell type composition in adult tissues is also sex-biased, perhaps adding another variable to be accounted for in the cardiac response if cell lineages have different sensitivities to hormone activity (14). Another complicating factor is that local production of steroids occurs in many somatic tissues and is not reflected by the levels of circulating hormones (22).

Although the fold differences in gene expression between males and females are sometimes discounted as small, the cumulative differences can significantly skew regulatory networks. Network analyses show substantial differences in regulatory structure between males and females (13, 23). Some transcription and epigenetic factors are differentially expressed in adult hearts, posing the question of how their dosage affects their downstream targets. On the other hand, many transcription factors, including the estrogen receptors ESR1 and ESR2 and the androgen receptor (AR), have sex-biased gene targets in other tissues, even if they are not differentially expressed themselves. This bias may be due to the epigenomic sex differences (24, 25), which would affect the accessibility of transcription factors to their cognate motifs. This raises the possibility that different sets of genes be regulated by the same transcription factor in male and female cells. Unfortunately, the few epigenomic studies in the heart are not sex-stratified (25), which hinders the ability to identify sex biases in regulatory factor accessibility to their recognition motifs. ChIP-seq studies on sex steroid receptors in male and female hearts would also be invaluable in identifying their genomic targets.

In addition to the above-mentioned complexities, commonly expressed genes in males and females may be targeted by different factors (13). Overall, the network structure differences resulting from the sum of these regulatory biases could have consequences for aging processes or cellular responses to stress or disease (26). This implies that the same disease in males and females can result from different pathways, indicating that therapeutic targets may be different as well. Little is known about the specific underlying mechanisms. However, they might include differences in the protein abundance or post-translational modification of transcription factors or differential availability of epigenetic co-factors, in addition to sex-biased access to recognition sites (27).

## Sex Differences in Cardiac Disease

In view of the sex differences in baseline cardiac function, it is not surprising that biological sex is a significant determinant in the development, presentation and progression of cardiovascular disease (28–30). The protective role of estrogen in women is well-known (17, 31–33). Men tend to develop cardiovascular disease earlier, whereas women develop these disorders later in life, accompanied by more comorbidities (34). Symptoms can also differ markedly between sexes. For example, women with myocardial infarction are less likely to present with chest pain than men, and more likely to have nausea, fatigue among other symptoms. Sex disparities in the impact of risk factors for cardiac disease are also evident (1, 35), and women have higher rates of adverse drug reactions (36, 37). Despite these glaring differences, the diagnostic criteria and the treatments proffered are usually the same for both men and women, based on clinical studies that over-represent males.

Although cardiac diseases resulting from genetic causes are not expected to vary by sex, they do in fact occur with differing prevalence between males and females in both humans and mice. For example, inherited hypertrophic cardiomyopathy due to mutations in sarcomeric proteins exhibit skewed incidence, indicating that sex-specific factors can compensate for the genetic defects and contribute to the penetrance of the disease (38). Hormonal differences are assumed to be responsible for these differences, but there are few mechanistic studies to support this notion, and little is known of the sex chromosome effects or developmental origins of these biases.

Genome-wide studies to identify risk alleles for cardiovascular disease have found sex differences (39–41). Gene-by-environment interactions, including the hormonal milieu, can detect variants with different magnitude or direction of association in the sexes. Associations with sex chromosome-linked gene variants have been identified, including the contribution of certain Y chromosome haplotypes to disease in both humans and mice (42–44). However, sex chromosome associations alone do not explain sex differences for highly polygenic cardiovascular traits and disease risk. Alternative models have been proposed to explain these observations. For example, the sex-dependent liability threshold model proposes that the sex with lower risk requires a greater number of risk alleles to exhibit the phenotype (45). Evidence for this model exists in neurodevelopmental disorders (46).

## The Impact of the Sex Chromosomes

After many years of pioneering studies, the awareness that sex chromosomes provide an intrinsic source of differential gene regulation is slowly spreading through the bloodstream of the scientific community (47–51). It has become patently clear that the inherent sexual inequality in expression of X and Y genes in non-gonadal tissues causes widespread sex differences in physiology and disease. In mouse models where chromosomal sex is genetically uncoupled from gonadal sex, all adult tissues exhibit sex-biased transcriptomes that can be directly attributed to the sex chromosome composition (52, 53). Strong evidence has emerged for *Kdm6a* and *Kdm5c* (Lysine-specific demethylase 6a and 5c), two X-linked genes and *Uty/Kdm6c* (Lysine-specific demethylase 6c), a Y-linked gene, as candidates for contributing to sex differences in mouse models of cancer, autoimmunity, metabolic disease and Alzheimer's (54–57). Evidence shows that the presence of two X chromosomes in female cardiovascular cells might act to increase susceptibility to ischemia/reperfusion injury (58, 59). Variants of Y chromosome loci also affect cardiac function (60), and the Y chromosome-linked genes TBL1Y and KDM5D have been reported to be involved in cardiac differentiation in human embryonic stem cells (61, 62). Mechanistic studies on the involvement of these genes in cardiac function are forthcoming, but the epigenetic functions of the encoded proteins predict that they may have widespread effects on the transcriptome. Identification of additional X and Y genes and their downstream targets in these models will shed light on their effects in the context of the heart, including how they act within sex-specific hormonal environments (63, 64).

## The Embryonic Origins of Sexual Dimorphism

One obvious gap in our understanding of sex differences is when they originate (65). As mentioned above, adult male and female hearts have substantial differences in their transcriptional and epigenetic profiles; however, it is not known whether any of these differences are established during embryogenesis. Sex differences at the molecular level have been woefully underexplored during early developmental stages under the assumption that embryogenesis is sex-neutral. Yet long-standing research shows that sex-biased gene expression is present before gonad formation, at stages when only the sex chromosome constitution differs (66–69). Sex chromosome-linked genes, including many dosage-sensitive transcription and epigenetic factors (70, 71), are expressed soon after fertilization, and subsequently affect the expression and epigenetic patterns of the autosomes. In fact, differences are already apparent in male and female preimplantation embryos and embryonic stem (ES) cells in mice and humans (72–75).

During early development, the sex chromosomes influence expression and epigenetic patterns by several mechanisms. First, Y chromosome-linked genes are only present in male cells. Second, female embryos have two active X chromosomes during a brief window before implantation, with the potential for establishing sex-specific epigenetic marks. After implantation, female embryos undergo X chromosome inactivation, a massive epigenetic event, that delays differentiation (76–78); this event has been hypothesized to alter the availability and composition of epigenetic complexes relative to male embryos (79). Third, although X chromosome inactivation equalizes most of the X-linked gene dosages between males and females, a subset of genes escape repression and are more highly expressed in female cells. Some X-linked genes escape in a tissue-specific manner (80–82), although early lineages have not yet been studied.

**Figure 2:**
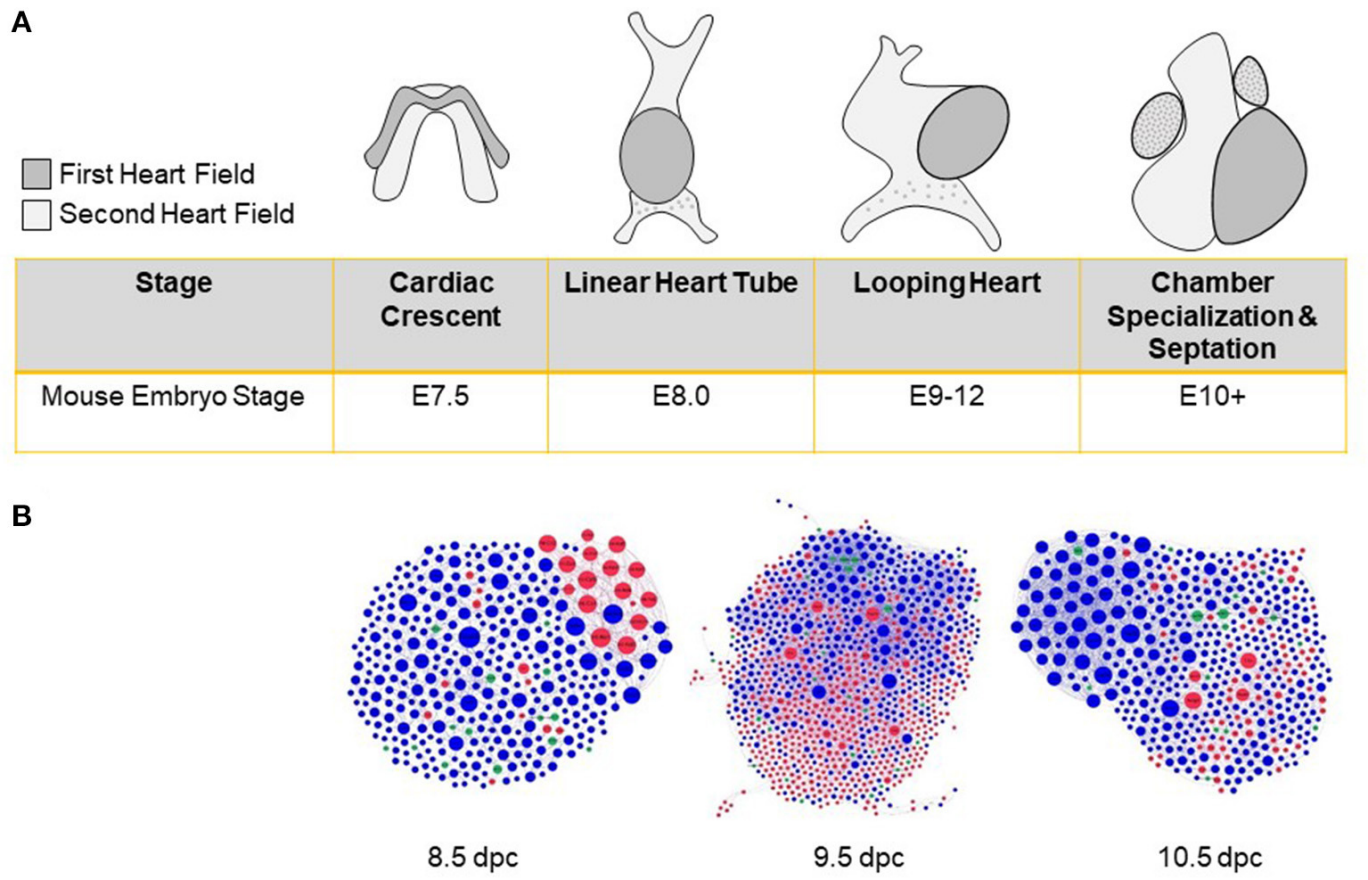
Sex biases in heart development before gonad formation. **(A)** Cardiac progenitors from the first and second heart fields establish the cardiac crescent during late gastrulation. Subsequent proliferation and differentiation of cells lead to the formation of a linear heart tube and cranial positioning of the atria. Remodeling then leads to chamber formation, septation, and valve development. **(B)** Protein-protein interaction networks were constructed from differentially expressed genes as assayed in single-cell RNA-seq experiments (84) and sex-stratified by us (83) for 8.5, 9.5, and 10.5 days *post-coitum* (dpc) hearts. Networks include sex-biased modules highlighted by red (female-enriched) and blue (male-enriched) nodes. Examples of sex-biased genes for 8.5, 9.5, and 10.5, respectively, include: Ctc1, Tbx2, Trim25 (female-biased); Km2a, Dusp3, Ube2c (male-biased).

As the first organ to develop, the heart exhibits sex-biased expression before gonadogenesis (83). Differentiation of cardiac precursors results in the convergence of male and female transcriptomes, but does not equalize all gene expression and epigenetic differences (83–85). Thus, male and female cellular identities are programmed into the genome well before sex hormones appear (86) (Figure 2). Notably, male and female human induced pluripotent stem cells adopt different developmental trajectories when differentiated into cardiac progenitors (87). A subset of genes that exhibit sex-specific expression in early cardiac development is also sex-biased in the adult heart (86), suggesting mechanisms that perpetuate these patterns.

Differentiation of the gonads and the secretion of sex hormones intersects with ongoing organogenesis and influences developmental patterns in all non-gonadal tissues, including the heart. These so-called “organizational” effects of hormones, which are permanent and distinct from the transient hormonal effects in adult tissues, have not been studied outside of the developing nervous system (88). It is unknown how the pre-established sex differences in the epigenomic landscape impact the genomic effects of sex steroids. Also unknown is whether hormones in turn reprogram the epigenome, reinforcing or counteracting the sex chromosome-dependent effects. Studies in non-mammalian models have shown that sex-biased expression waxes and wanes during development (89), but detailed time-course analyses in mammals have not been done.

Since many diseases with developmental origins display sex biases (90, 91), it is important to study the role of sex-biased gene expression—and any underlying mechanisms—during embryogenesis. More specifically, many congenital heart defects exhibit unexplained sex biases in presentation, mortality and morbidity (92–95). The fact that 45% of Turner Syndrome patients (X chromosome monosomy) suffer from congenital heart defects underscores the role of the sex chromosomes in embryogenesis (96). We must identify the relevant genes and their effects on the well-established cardiac development network (97–100).

## The Developmental Origins of Cardiac Disease

There is unequivocal evidence that the prenatal environment can have long-term consequences on health (101). Compelling data suggest that some cardiac diseases may be programmed *in utero* (102–104). Epigenetic mechanisms record developmental and environmental events that can be translated later into expression patterns. In the same vein, sex-specific information resulting from sex-biased transcription or epigenetic factors during development may be stored in the epigenome, with consequences on later life traits (91). Epidemiological evidence abounds from both human and animal studies showing that male and female embryos are not equivalent. Male embryos are more vulnerable to developmental disorders than females, partly because of fetal-maternal interactions and possibly due to sex differences in developmental trajectories. Moreover, the placenta, which originates mainly from fetal lineages and has the sex chromosome composition of the embryo, also has a role in the susceptibility to gestational insults (105–107).

Despite the faster growth rates of male embryos, organ systems develop more slowly in male fetuses, potentially making them less adaptable to adverse uterine environments (108, 109). Indeed, a range of maternal conditions consistently increases cardiovascular disease risk more in males than in females (110, 111). Perhaps cardiogenesis in the female embryos is more resilient because of the later structural and hemodynamic demands of pregnancy (112).

## Evolutionary Considerations for Sex Differences Across Developmental Stages

The discovery of widespread sex-biased gene expression raises questions about the evolutionary forces that drive these differences in all somatic tissues, including the heart, and intriguingly, across all developmental stages. Evolutionary theory can provide valuable insights into the origins of sex differences for normal physiological traits, such as sex biases in longevity, as well as for disease prevalence (113). In turn, elucidating the mechanisms and dynamics of sex biases in humans can inform the evolutionary frameworks for explaining conflicting selection pressures on males and females.

Sexual conflict results from the divergent fitness interests of males and females. The vast majority of the genes encoding traits shared by males and females is common, but some genes can have different optimal expression levels for each sex, leading to suboptimal levels for both (114–116). Sex-biased gene expression can indicate the existence of regulatory mechanisms to offset suboptimal levels by allowing each sex to approximate its fitness optimum (117, 118). For example, *cis*- or trans-regulatory mechanisms, sex-dependent methylation, differentially spliced transcript forms, or gene duplication with the emergence of sex-specific regulation for each duplicate, are all mechanisms that can resolve sexual conflict by uncoupling genetic architecture between males and females (117). In mammals, with their greatly expanded families of transcription factors, it would be interesting to determine whether paralogs harbor divergent regulatory sequences that result in sex-specific expression, with the concomitant cascading effect on their target genes.

The relationship between sexual antagonism and sex-biased gene expression is still uncertain. It is likely that not all sex-biased genes are relevant (89) and it is possible that genes expressed at equivalent levels in both sexes can result in differing phenotypic effects. In addition, genes underlying traits undergoing sex-specific selection can be at different evolutionary stages (113). Additional complications arise in considering complex traits resulting from many loci of small effects.

Forces generating sex-biased expression differences are likely to be most prominent in adults, when reproductive interests diverge. However, the studies in mouse and human embryonic stem cells and embryos indicate that sex-biased expression is present across all developmental stages. In addition, the sex bias of a gene can vary in a developmental stage- or tissue-specific manner. If we envision sexual differentiation more broadly as a progressive developmental process beginning soon after fertilization and encompassing non-gonadal as well as gonadal tissues, sex-specific selection pressures might operate throughout embryogenesis and shift across the lifespan (119).

These discoveries prompt several questions: (1) how are sex biases related to the differential response to the intrauterine environment exhibited by male and female embryos? (2) which sex-biased genes truly encode sexually dimorphic traits because of sex-specific selection? (3) how do sex-biased expression patterns relate to adult-stage sex differences? Answers to these questions in the specific case of cardiac development and adult cardiac phenotypes will lead to a more profound understanding of how sex affects cardiovascular health and disease.

One useful framework for considering sex differences during embryogenesis and their potential contribution to later stage health disparities is adaptive developmental plasticity (120). During fetal development, environmental cues can elicit responses aimed at interpreting the present conditions and optimizing post-natal strategies for maximal fitness by predicting future conditions. These responses are mediated in part by epigenetic mechanisms (109, 121). This framework implies certain trade-offs in long-lived species such as humans, where the potential for a mismatch between pre- and post-natal conditions can contribute to disease risk (122). Although sex differences have not been formally incorporated into these evolutionary models, evidence of sex-specific developmental trade-offs will likely be considered in future work (123).

## Unanswered Questions and Future Directions

It has been 20 years since the Institute of Medicine of the National Academies of Science published an exhaustive examination of how sex and gender contribute to human health and disease (124). Despite this, research to discern the mechanisms that explain sex differences is still in its infancy. Nowhere is this more evident than in developmental biology, which has circumscribed the study of sex differences to the process of gonadal differentiation, thereby overlooking the effects of sex chromosome-linked genes in early embryogenesis.

The advent of genomic technologies has certainly adrenalized the study of sex differences. However, many unanswered questions remain regarding whether sex differences in specific cardiac phenotypes are due to hormones, genetic sex, or sex-hormone interactions. Also unknown is if the target genes of sex hormones differ in each sex. In this regard, there is a need for studies comparing the effects of specific sex hormones on both male and female hearts, instead of focusing exclusively on how androgens act in males and estrogens in females. With the expansion of the transgendered population, cross-hormone therapy has become a standard treatment. To identify and manage the potential risks facing these patients, studies with animal models administering estrogen in males and androgens in females have been performed (125). However, these experiments do not elucidate how normal estrogen levels in males differ in effects from normal estrogen levels in females. Thus, further investigation is also required to fully understand the programs controlled by the sex chromosomes in the heart. Moreover, mitochondrial function differs between male and female hearts, but the molecular basis of these differences is unknown (24, 126).

Not all sex-biased genes are likely to be involved in the functional differences between male and female hearts, so it is critical to establish the exact relationship between RNA abundance and phenotypes. Some differential expression may have evolved to compensate for rather than enhance the disparities. Studies in a broader spectrum of model organisms is essential to understand the evolutionary principles that lead to sex-specific transcriptomes.

There are some caveats to the use of transcriptomes for studying sexual dimorphism. RNA levels are an incomplete indicator without complementing them with proteomic analyses. Biases in splice forms have not been thoroughly investigated and could be a factor in sex-specific activities, as reported previously in primates, including humans (127). Regulatory factors present at equal levels should not be discounted, as post-translational modifications could also be dependent on sex.

There are still very few studies on the timing and persistence of sex-biased gene expression during heart development. The identities of sex chromosome-linked genes expressed from early embryogenesis, and the mutual interactions between the sex chromosomes and autosomes are unknown. A thorough understanding of the interactions of sex chromosomes and hormones throughout cardiac development is also needed. Whether the appearance of sex hormones erases or enhances pre-established differences, it will be interesting to understand the underlying mechanisms. It is also important to determine the effects of hormones on the early stages of heart organization.

Many genes are expressed with less than two-fold differences between males and females (11, 14, 72, 83). Although these differences are generally discounted as insignificant, their impact can propagate along the regulatory networks that characterize cell states, shifting phenotypic outcomes. Network modeling has identified differential targeting of genes and revealed regulatory differences that could be important under conditions of stress, age or in response to therapies, even when baseline expression levels are only slightly dissimilar (11, 128, 129). The use of the continuously evolving systems biology tools will allow us to analyze the aggregate pattern of sex differences in expression to uncover sex-specific modules in global networks.

In conclusion, there is much to be learned from basic studies of sex differences in the heart across all life stages. We have only begun to fully understand the significance of early developmental sex differences. Detailed mechanistic studies will enable us to eventually develop more selective, sex-specific interventions in cardiovascular disease.

## Author Contributions

DD and PN contributed to bibliography compilation and review of the manuscript content. NE wrote and reviewed the manuscript. All authors approved the final version of the manuscript.

## Conflict of Interest

The authors declare that the research was conducted in the absence of any commercial or financial relationships that could be construed as a potential conflict of interest.

## References

[1] Mauvais-Jarvis F Bairey Merz N Barnes PJ Brinton RD Carrero JJ DeMeo DL. Sex and gender: modifiers of health, disease, and medicine. Lancet. (2020) 396:565–82. 10.1016/S0140-6736(20)31561-0 32828189 PMC7440877

[2] Clayton JA. Applying the new SABV (sex as a biological variable) policy to research and clinical care. Physiol Behav. (2018) 187:2–5. 10.1016/j.physbeh.2017.08.012 28823546

[3] Geller SE Koch A Pellettieri B Carnes M. Inclusion, analysis, and reporting of sex and race/ethnicity in clinical trials: have we made progress? J Womens Health. (2011) 20:315–20. 10.1089/jwh.2010.2469 21351877 PMC3058895

[4] Blenck CL Harvey PA Reckelhoff JF Leinwand LA. The importance of biological sex and estrogen in rodent models of cardiovascular health and disease. Circ Res. (2016) 118:1294–312. 10.1161/CIRCRESAHA.116.307509 27081111 PMC4834858

[5] Ventura-Clapier R Dworatzek E Seeland U Kararigas G Arnal JF Brunelleschi S. Sex in basic research: concepts in the cardiovascular field. Cardiovasc Res. (2017) 113:711–24. 10.1093/cvr/cvx066 28472454

[6] Coronado MJ Fairweather D Bruno KA. Sex determines cardiac myocyte stretch and relaxation. Circ Cardiovasc Genet. 10:e001950. 10.1161/CIRCGENETICS.117.001950 29030406 PMC5685666

[7] Norheim F Hasin-Brumshtein Y Vergnes L Chella Krishnan K Pan C Seldin MM. Gene-by-sex interactions in mitochondrial functions and cardio-metabolic traits. Cell Metab. (2019) 29:932–49 e4. 10.1016/j.cmet.2018.12.013 30639359 PMC6447452

[8] Kararigas G Bito V Tinel H Becher E Baczko I Knosalla C. Transcriptome characterization of estrogen-treated human myocardium identifies myosin regulatory light chain interacting protein as a sex-specific element influencing contractile function. J Am Coll Cardiol. (2012) 59:410–7. 10.1016/j.jacc.2011.09.054 22261164

[9] Singmann P Shem-Tov D Wahl S Grallert H Fiorito G Shin SY. Characterization of whole-genome autosomal differences of DNA methylation between men and women. Epigenetics Chromatin. (2015) 8:43. 10.1186/s13072-015-0035-3 26500701 PMC4615866

[10] Mayne BT Bianco-Miotto T Buckberry S Breen J Clifton V Roberts CT. Large scale gene expression meta-analysis reveals tissue-specific, sex-biased gene expression in humans. Front Genet. (2016) 7:183. 10.3389/fgene.2016.00183 27790248 PMC5062749

[11] Gershoni M Pietrokovski S. The landscape of sex-differential transcriptome and its consequent selection in human adults. BMC Biol. (2017) 15:7. 10.1186/s12915-017-0352-z 28173793 PMC5297171

[12] InanlooRahatloo K Liang G Vo D Ebert A Nguyen I Nguyen PK. Sex-based differences in myocardial gene expression in recently deceased organ donors with no prior cardiovascular disease. PLoS ONE. (2017) 12:e0183874. 10.1371/journal.pone.0183874 28850583 PMC5574577

[13] Lopes-Ramos CM Chen CY Kuijjer ML Paulson JN Sonawane AR Fagny M. Sex differences in gene expression and regulatory networks across 29 human tissues. Cell Rep. (2020) 31:107795. 10.1016/j.celrep.2020.107795 32579922 PMC7898458

[14] Oliva M Munoz-Aguirre M Kim-Hellmuth S Wucher V Gewirtz ADH Cotter DJ. The impact of sex on gene expression across human tissues. Science. (2020) 369:eaba3066. 10.1126/science.aba3066 32913072 PMC8136152

[15] Synnergren J Vukusic K Donnes P Jonsson M Lindahl A Dellgren G. Transcriptional sex and regional differences in paired human atrial and ventricular cardiac biopsies collected *in vivo*. Physiol Genomics. (2020) 52:110–20. 10.1152/physiolgenomics.00036.2019 31869284

[16] Morselli E Santos RS Criollo A Nelson MD Palmer BF Clegg DJ. The effects of oestrogens and their receptors on cardiometabolic health. Nat Rev Endocrinol. (2017) 13:352–64. 10.1038/nrendo.2017.12 28304393

[17] Clegg D Hevener AL Moreau KL Morselli E Criollo A Van Pelt RE. Sex hormones and cardiometabolic health: role of estrogen and estrogen receptors. Endocrinology. (2017) 158:1095–105. 10.1210/en.2016-1677 28323912 PMC6283431

[18] Machuki JO Zhang HY Harding SE Sun H. Molecular pathways of oestrogen receptors and beta-adrenergic receptors in cardiac cells: recognition of their similarities, interactions and therapeutic value. Acta Physiol. (2018) 222:e12978. 10.1111/apha.12978 PMC5813217 28994249

[19] Isensee J Witt H Pregla R Hetzer R Regitz-Zagrosek V Noppinger PR. Sexually dimorphic gene expression in the heart of mice and men. J Mol Med. (2008) 86:61–74. 10.1007/s00109-007-0240-z 17646949 PMC2755745

[20] Trexler CL Odell AT Jeong MY Dowell RD Leinwand LA. Transcriptome and functional profile of cardiac myocytes is influenced by biological sex. Circ Cardiovasc Genet. (2017) 10:e001770. 10.1161/CIRCGENETICS.117.001770 29030402 PMC5679409

[21] Pugach EK Blenck CL Dragavon JM Langer SJ Leinwand LA. Estrogen receptor profiling and activity in cardiac myocytes. Mol Cell Endocrinol. (2016) 431:62–70. 10.1016/j.mce.2016.05.004 27164442 PMC4899180

[22] Miller LR Marks C Becker JB Hurn PD Chen WJ Woodruff T. Considering sex as a biological variable in preclinical research. FASEB J. (2017) 31:29–34. 10.1096/fj.201600781r 27682203 PMC6191005

[23] Arnold AP van Nas A Lusis AJ. Systems biology asks new questions about sex differences. Trends Endocrinol Metab. (2009) 20:471–6. 10.1016/j.tem.2009.06.007 19783453 PMC2787703

[24] Silkaitis K Lemos B. Sex-biased chromatin and regulatory cross-talk between sex chromosomes, autosomes, and mitochondria. Biol Sex Differ. (2014) 5:2. 10.1186/2042-6410-5-2 24422881 PMC3907150

[25] Hartman RJG Huisman SE den Ruijter HM. Sex differences in cardiovascular epigenetics-a systematic review. Biol Sex Differ. (2018) 9:19. 10.1186/s13293-018-0180-z 29792221 PMC5966883

[26] Theodoris CV Zhou P Liu L Zhang Y Nishino T Huang Y. Network-based screen in iPSC-derived cells reveals therapeutic candidate for heart valve disease. Science. (2020) 26:eabd0724. 10.1126/science.abd0724 33303684 PMC7880903

[27] Yin Y Morgunova E Jolma A Kaasinen E Sahu B Khund-Sayeed S. Impact of cytosine methylation on DNA binding specificities of human transcription factors. Science. (2017) 356:eaaj2239. 10.1126/science.aaj2239 28473536 PMC8009048

[28] Maric-Bilkan C Arnold AP Taylor DA Dwinell M Howlett SE Wenger N. Report of the National Heart, Lung, and Blood Institute Working Group on sex differences research in cardiovascular disease: scientific questions and challenges. Hypertension. (2016) 67:802–7. 10.1161/HYPERTENSIONAHA.115.06967 26975706 PMC4833564

[29] Humphries KH Izadnegahdar M Sedlak T Saw J Johnston N Schenck-Gustafsson K. Sex differences in cardiovascular disease - impact on care and outcomes. Front Neuroendocrinol. (2017) 46:46. 10.1016/j.yfrne.2017.04.001 28428055 PMC5506856

[30] Regitz-Zagrosek V Kararigas G. Mechanistic pathways of sex differences in cardiovascular disease. Physiol Rev. (2017) 97:1–37. 10.1152/physrev.00021.2015 27807199

[31] Pare G Krust A Karas RH Dupont S Aronovitz M Chambon P. Estrogen receptor-alpha mediates the protective effects of estrogen against vascular injury. Circ Res. (2002) 90:1087–92. 10.1161/01.RES.0000021114.92282.FA 12039798

[32] Mozaffarian D Benjamin EJ Go AS Arnett DK Blaha MJ Cushman M. Heart disease and stroke statistics–2015 update: a report from the American Heart Association. Circulation. (2015) 131:e29–322. 25520374 10.1161/CIR.0000000000000152

[33] Schuster I Mahmoodzadeh S Dworatzek E Jaisser F Messaoudi S Morano I. Cardiomyocyte-specific overexpression of oestrogen receptor beta improves survival and cardiac function after myocardial infarction in female and male mice. Clin Sci. (2016) 130:365–76. 10.1042/CS20150609 26608078

[34] den Ruijter HM Haitjema S Asselbergs FW Pasterkamp G. Sex matters to the heart: a special issue dedicated to the impact of sex related differences of cardiovascular diseases. Atherosclerosis. (2015) 241:205–7. 10.1016/j.atherosclerosis.2015.05.003 26003338

[35] Gerdts E Regitz-Zagrosek V. Sex differences in cardiometabolic disorders. Nat Med. (2019) 25:1657–66. 10.1038/s41591-019-0643-8 31700185

[36] Heinrich J Gahart MT Rowe EJ Bradley L. Drug Safety: Most Drugs Withdrawn in Recent Years Had Greater Health Risks for Women. GAO (2001). Available online at: https://www.gao.gov/assets/100/90642.pdf

[37] Yu Y Chen J Li D Wang L Wang W Liu H. Systematic analysis of adverse event reports for sex differences in adverse drug events. Sci Rep. (2016) 6:24955. 10.1038/srep24955 27102014 PMC4840306

[38] Meyer S van der Meer P van Tintelen JP van den Berg MP. Sex differences in cardiomyopathies. Eur J Heart Fail. (2014) 16:238–47. 10.1002/ejhf.15 24464619

[39] Liu LY Schaub MA Sirota M Butte AJ. Sex differences in disease risk from reported genome-wide association study findings. Hum Genet. (2012) 131:353–64. 10.1007/s00439-011-1081-y 21858542 PMC3260375

[40] Rawlik K Canela-Xandri O Tenesa A. Evidence for sex-specific genetic architectures across a spectrum of human complex traits. Genome Biol. (2016) 17:166. 10.1186/s13059-016-1025-x 27473438 PMC4965887

[41] Traglia M Bseiso D Gusev A Adviento B Park DS Mefford JA. Genetic mechanisms leading to sex differences across common diseases and anthropometric traits. Genetics. (2017) 205:979–92. 10.1534/genetics.116.193623 27974502 PMC5289864

[42] Charchar FJ Bloomer LD Barnes TA Cowley MJ Nelson CP Wang Y. Inheritance of coronary artery disease in men: an analysis of the role of the Y chromosome. Lancet. (2012) 379:915–22. 10.1016/S0140-6736(12)61061-7 22325189 PMC3314981

[43] Bloomer LD Nelson CP Eales J Denniff M Christofidou P Debiec R. Male-specific region of the Y chromosome and cardiovascular risk: phylogenetic analysis and gene expression studies. Arterioscler Thromb Vasc Biol. (2013) 33:1722–7. 10.1161/ATVBAHA.113.301608 23640493

[44] Maan AA Eales J Akbarov A Rowland J Xu X Jobling MA. The Y chromosome: a blueprint for men's health? Eur J Hum Genet. (2017) 25:1181–8. 10.1038/ejhg.2017.128 28853720 PMC5643963

[45] Carter CO Evans KA. Inheritance of congenital pyloric stenosis. J Med Genet. (1969) 6:233–54. 10.1136/jmg.6.3.233 5345095 PMC1468738

[46] Jacquemont S Coe BP Hersch M Duyzend MH Krumm N Bergmann S. A higher mutational burden in females supports a “female protective model” in neurodevelopmental disorders. Am J Hum Genet. (2014) 94:415–25. 10.1016/j.ajhg.2014.02.001 24581740 PMC3951938

[47] Kukurba KR Parsana P Balliu B Smith KS Zappala Z Knowles DA. Impact of the X chromosome and sex on regulatory variation. Genome Res. (2016) 26:768–77. 10.1101/gr.197897.115 27197214 PMC4889977

[48] Rigby N Kulathinal RJ. Genetic architecture of sexual dimorphism in humans. J Cell Physiol. (2015) 230:2304–10. 10.1002/jcp.24979 25740260

[49] Abramowitz LK Olivier-Van Stichelen S Hanover JA. Chromosome imbalance as a driver of sex disparity in disease. J Genomics. (2014) 2:77–88. 10.7150/jgen.8123 25031659 PMC4091450

[50] Ho B Greenlaw K Al Tuwaijri A Moussette S Martínez F Giorgio E. X chromosome dosage and presence of SRY shape sex-specific differences in DNA methylation at an autosomal region in human cells. Biol Sex Differ. (2018) 9:10. 10.1186/s13293-018-0169-7 29463315 PMC5819645

[51] Raznahan A Parikshak NN Chandran V Blumenthal JD Clasen LS Alexander-Bloch AF. Sex-chromosome dosage effects on gene expression in humans. Proc Natl Acad Sci USA. (2018) 115:7398–403. 10.1073/pnas.1802889115 29946024 PMC6048519

[52] Burgoyne PS Arnold AP. A primer on the use of mouse models for identifying direct sex chromosome effects that cause sex differences in non-gonadal tissues. Biol Sex Differ. (2016) 7:68. 10.1186/s13293-016-0115-5 27999654 PMC5154145

[53] Wijchers PJ Yandim C Panousopoulou E Ahmad M Harker N Saveliev A. Sexual dimorphism in mammalian autosomal gene regulation is determined not only by Sry but by sex chromosome complement as well. Dev Cell. (2010) 19:477–84. 10.1016/j.devcel.2010.08.005 20833369

[54] Arnold AP Disteche CM. Sexual inequality in the cancer cell. Cancer Res. (2018) 78:5504–5. 10.1158/0008-5472.CAN-18-2219 30275051 PMC6204258

[55] Link JC Wiese CB Chen X Avetisyan R Ronquillo E Ma F. X chromosome dosage of histone demethylase KDM5C determines sex differences in adiposity. J Clin Invest. (2020) 130:5688–702. 10.1172/JCI140223 32701509 PMC7598065

[56] Itoh Y Golden LC Itoh N Matsukawa MA Ren E Tse V. The X-linked histone demethylase Kdm6a in CD4+ T lymphocytes modulates autoimmunity. J Clin Invest. (2019) 129:3852–63. 10.1172/JCI126250 31403472 PMC6715385

[57] Davis EJ Broestl L Abdulai-Saiku S Worden K Bonham LW Minones-Moyano E. A second X chromosome contributes to resilience in a mouse model of Alzheimer's disease. Sci Transl Med. (2020) 12:eaaz5677. 10.1126/scitranslmed.aaz5677 32848093 PMC8409261

[58] Li J Chen X McClusky R Ruiz-Sundstrom M Itoh Y Umar S. The number of X chromosomes influences protection from cardiac ischaemia/reperfusion injury in mice: one X is better than two. Cardiovasc Res. (2014) 102:375–84. 10.1093/cvr/cvu064 24654234 PMC4030514

[59] Arnold AP Cassis LA Eghbali M Reue K Sandberg K. Sex hormones and sex chromosomes cause sex differences in the development of cardiovascular diseases. Arterioscler Thromb Vasc Biol. (2017) 37:746–56. 10.1161/ATVBAHA.116.307301 28279969 PMC5437981

[60] Praktiknjo SD Picard S Deschepper CF. Comparisons of chromosome Y-substituted mouse strains reveal that the male-specific chromosome modulates the effects of androgens on cardiac functions. Biol Sex Differ. (2016) 7:61. 10.1186/s13293-016-0116-4 27980711 PMC5143463

[61] Meyfour A Ansari H Pahlavan S Mirshahvaladi S Rezaei-Tavirani M Gourabi H. Y chromosome missing protein, TBL1Y, may play an important role in cardiac differentiation. J Proteome Res. (2017) 16:4391–402. 10.1021/acs.jproteome.7b00391 28853286

[62] Meyfour A Pahlavan S Ansari H Baharvand H Salekdeh GH. Down-regulation of a male-specific H3K4 demethylase, KDM5D, impairs cardiomyocyte differentiation. J Proteome Res. (2019) 18:4277–82. 10.1021/acs.jproteome.9b00395 31560558

[63] Deschepper CF. Regulatory effects of the Uty/Ddx3y locus on neighboring chromosome Y genes and autosomal mRNA transcripts in adult mouse non-reproductive cells. Sci Rep. (2020) 10:14900. 10.1038/s41598-020-71447-3 32913328 PMC7484786

[64] Meyfour A Pooyan P Pahlavan S Rezaei-Tavirani M Gourabi H Baharvand H. Chromosome-centric human proteome project allies with developmental biology: a case study of the role of y chromosome genes in organ development. J Proteome Res. (2017) 16:4259–72. 10.1021/acs.jproteome.7b00446 28914051

[65] Engel N. Sex differences in early embryogenesis: inter-chromosomal regulation sets the stage for sex-biased gene networks: the dialogue between the sex chromosomes and autosomes imposes sexual identity soon after fertilization. Bioessays. (2018) 40:1800073. 10.1002/bies.201800073 29943439

[66] Burgoyne PS Thornhill AR Boudrean SK Darling SM Bishop CE Evans EP. The genetic basis of XX-XY differences present before gonadal sex differentiation in the mouse. Philos Trans R Soc Lond B Biol Sci. (1995) 350:253–60; discussion: 260–1. 10.1098/rstb.1995.0159 8570689

[67] Berletch JB Deng X Nguyen DK Disteche CM. Female bias in Rhox6 and 9 regulation by the histone demethylase KDM6A. PLoS Genet. (2013) 9:e1003489. 10.1371/journal.pgen.1003489 23658530 PMC3642083

[68] Lowe R Gemma C Rakyan VK Holland ML. Sexually dimorphic gene expression emerges with embryonic genome activation and is dynamic throughout development. BMC Genomics. (2015) 16:295. 10.1186/s12864-015-1506-4 25888192 PMC4410000

[69] Bermejo-Alvarez P Rizos D Lonergan P Gutierrez-Adan A. Transcriptional sexual dimorphism during preimplantation embryo development and its consequences for developmental competence and adult health and disease. Reproduction. (2011) 141:563–70. 10.1530/REP-10-0482 21339284

[70] Bellott DW Hughes JF Skaletsky H Brown LG Pyntikova T Cho TJ. Mammalian Y chromosomes retain widely expressed dosage-sensitive regulators. Nature. (2014) 508:494–9. 10.1038/nature13206 24759411 PMC4139287

[71] Disteche CM. Dosage compensation of the sex chromosomes and autosomes. Semin Cell Dev Biol. (2016) 56:9–18. 10.1016/j.semcdb.2016.04.013 27112542 PMC4955796

[72] Werner R Schultz BM Huhn JM Jelinek J Madzo J Engel N. Sex chromosomes drive gene expression and regulatory dimorphisms in mouse embryonic stem cells. Biol Sex Differ. (2017) 8:28. 10.1186/s13293-017-0150-x 28818098 PMC5561606

[73] Ronen D Benvenisty N. Sex-dependent gene expression in human pluripotent stem cells. Cell Rep. (2014) 8:923–32. 10.1016/j.celrep.2014.07.013 25127145

[74] Chen G Schell JP Benitez JA Petropoulos S Yilmaz M Reinius B. Single-cell analyses of X chromosome inactivation dynamics and pluripotency during differentiation. Genome Res. (2016) 26:1342–54. 10.1101/gr.201954.115 27486082 PMC5052059

[75] Marks H Kalkan T Menafra R Denissov S Jones K Hofemeister H. The transcriptional and epigenomic foundations of ground state pluripotency. Cell. (2012) 149:590–604. 10.1016/j.cell.2012.03.026 22541430 PMC3398752

[76] Schulz EG Meisig J Nakamura T Okamoto I Sieber A Picard C. The two active X chromosomes in female ESCs block exit from the pluripotent state by modulating the ESC signaling network. Cell Stem Cell. (2014) 14:203–16. 10.1016/j.stem.2013.11.022 24506884

[77] Payer B Rosenberg M Yamaji M Yabuta Y Koyanagi-Aoi M Hayashi K. Tsix RNA and the germline factor, PRDM14, link X reactivation and stem cell reprogramming. Mol Cell. (2013) 52:805–18. 10.1016/j.molcel.2013.10.023 24268575 PMC3950835

[78] Del Rosario BC Del Rosario AM Anselmo A Wang PI Sadreyev RI Lee JT. Genetic intersection of Tsix and Hedgehog signaling during the initiation of X-chromosome inactivation. Dev Cell. (2017) 43:359–71 e6. 10.1016/j.devcel.2017.09.027 29107559 PMC5701747

[79] Arnold AP Reue K Eghbali M Vilain E Chen X Ghahramani N. The importance of having two X chromosomes. Philos Trans R Soc Lond B Biol Sci. (2016) 371:20150113. 10.1098/rstb.2015.0113 26833834 PMC4785899

[80] Berletch JB Ma W Yang F Shendure J Noble WS Disteche CM. Escape from X inactivation varies in mouse tissues. PLoS Genet. (2015) 11:e1005079. 10.1371/journal.pgen.1005079 25785854 PMC4364777

[81] Balaton BP Brown CJ. Escape artists of the X chromosome. Trends Genet. (2016) 32:348–59. 10.1016/j.tig.2016.03.007 27103486

[82] Tukiainen T Villani AC Yen A Rivas MA Marshall JL Satija R. Landscape of X chromosome inactivation across human tissues. Nature. (2017) 550:244–8. 10.1038/nature24265 29022598 PMC5685192

[83] Deegan DF Karbalaei R Madzo J Kulathinal RJ Engel N. The developmental origins of sex-biased expression in cardiac development. Biol Sex Differ. (2019) 10:46. 10.1186/s13293-019-0259-1 31488212 PMC6727560

[84] Li G Xu A Sim S Priest JR Tian X Khan T. Transcriptomic profiling maps anatomically patterned subpopulations among single embryonic cardiac cells. Dev Cell. (2016) 39:491–507. 10.1016/j.devcel.2016.10.014 27840109 PMC5130110

[85] DeLaughter DM Bick AG Wakimoto H McKean D Gorham JM Kathiriya IS . Single-cell resolution of temporal gene expression during heart development. Dev Cell. (2016) 39:480–90. 10.1016/j.devcel.2016.10.001 27840107 PMC5198784

[86] Deegan DF Engel N. Sexual dimorphism in the age of genomics: how, when, where. Front Cell Dev Biol. (2019) 7:186. 10.3389/fcell.2019.00186 31552249 PMC6743004

[87] D'Antonio-Chronowska A Donovan MKR Young Greenwald WW Nguyen JP Fujita K Hashem S. Association of human iPSC gene signatures and X chromosome dosage with two distinct cardiac differentiation trajectories. Stem Cell Rep. (2019) 13:924–38. 10.1016/j.stemcr.2019.09.011 31668852 PMC6895695

[88] Arnold AP. The organizational-activational hypothesis as the foundation for a unified theory of sexual differentiation of all mammalian tissues. Horm Behav. (2009) 55:570–8. 10.1016/j.yhbeh.2009.03.011 19446073 PMC3671905

[89] Mank JE. Population genetics of sexual conflict in the genomic era. Nat Rev Genet. (2017) 18:721–30. 10.1038/nrg.2017.83 29062057

[90] Deciphering Developmental Disorders S. Large-scale discovery of novel genetic causes of developmental disorders. Nature. (2015) 519:223–8. 10.1038/nature14135 25533962 PMC5955210

[91] Sundrani DP Roy SS Jadhav AT Joshi SR. Sex-specific differences and developmental programming for diseases in later life. Reprod Fertil Dev. (2017) 29:2085–99. 10.1071/RD16265 28380326

[92] Somerville J. The denolin lecture: the woman with congenital heart disease. Eur Heart J. (1998) 19:1766–75. 10.1053/euhj.1998.1204 9886718

[93] Mercuro G Bassareo PP Mariucci E Deidda M Zedda AM Bonvicini M. Sex differences in congenital heart defects and genetically induced arrhythmias. J Cardiovasc Med. (2014) 15:855–63. 10.2459/JCM.0b013e32835ec828 23422886

[94] Engelfriet P Mulder BJ. Gender differences in adult congenital heart disease. Neth Heart J. (2009) 17:414–7. 10.1007/BF03086294 19949709 PMC2779477

[95] Hoang TT Goldmuntz E Roberts AE Chung WK Kline JK Deanfield JE. The congenital heart disease genetic network study: cohort description. PLoS ONE. (2018) 13:e0191319. 10.1371/journal.pone.0191319 29351346 PMC5774789

[96] Bondy CA. Congenital cardiovascular disease in Turner syndrome. Congenit Heart Dis. (2008) 3:2–15. 10.1111/j.1747-0803.2007.00163.x 18373744

[97] Evans SM Yelon D Conlon FL Kirby ML. Myocardial lineage development. Circ Res. (2010) 107:1428–44. 10.1161/CIRCRESAHA.110.227405 21148449 PMC3073310

[98] Srivastava D. Making or breaking the heart: from lineage determination to morphogenesis. Cell. (2006) 126:1037–48. 10.1016/j.cell.2006.09.003 16990131

[99] Jain R Epstein JA. Competent for commitment: you've got to have heart! Genes Dev. (2018) 32:4–13. 10.1101/gad.308353.117 29440224 PMC5828393

[100] Bruneau BG. Signaling and transcriptional networks in heart development and regeneration. Cold Spring Harb Perspect Biol. (2013) 5:a008292. 10.1101/cshperspect.a008292 23457256 PMC3578359

[101] Fleming TP Velazquez MA Eckert JJ. Embryos, DOHaD and David Barker. J Dev Orig Health Dis. (2015) 6:377–83. 10.1017/S2040174415001105 25952250

[102] Barker DJ Bagby SP. Developmental antecedents of cardiovascular disease: a historical perspective. J Am Soc Nephrol. (2005) 16:2537–44. 10.1681/ASN.2005020160 16049070

[103] Thornburg KL. The programming of cardiovascular disease. J Dev Orig Health Dis. (2015) 6:366–76. 10.1017/S2040174415001300 26173733 PMC7587080

[104] Svoboda LK Wang K Cavalcante RG Neier K Colacino JA Sartor MA. Sex-specific programming of cardiac DNA METHYLATION BY DEVELOPMENTAL PHTHALATE EXPOSURE. Epigenet Insights. (2020) 13:2516865720939971. 10.1177/2516865720939971 32864567 PMC7430087

[105] Nugent BM Bale TL. The omniscient placenta: metabolic and epigenetic regulation of fetal programming. Front Neuroendocrinol. (2015) 39:28–37. 10.1016/j.yfrne.2015.09.001 26368654 PMC4681645

[106] Bale TL. The placenta and neurodevelopment: sex differences in prenatal vulnerability. Dialogues Clin Neurosci. (2016) 18:459–64. 10.31887/DCNS.2016.18.4/tbale 28179817 PMC5286731

[107] Gabory A Roseboom TJ Moore T Moore LG Junien C. Placental contribution to the origins of sexual dimorphism in health and diseases: sex chromosomes and epigenetics. Biol Sex Differ. (2013) 4:5. 10.1186/2042-6410-4-5 23514128 PMC3618244

[108] Dipietro JA Voegtline KM. The gestational foundation of sex differences in development and vulnerability. Neuroscience. (2017) 342:4–20. 10.1016/j.neuroscience.2015.07.068 26232714 PMC4732938

[109] Reichetzeder C Dwi Putra SE Li J Hocher B. Developmental origins of disease - crisis precipitates change. Cell Physiol Biochem. (2016) 39:919–38. 10.1159/000447801 27513464

[110] Muralimanoharan S Li C Nakayasu ES Casey CP Metz TO Nathanielsz PW. Sexual dimorphism in the fetal cardiac response to maternal nutrient restriction. J Mol Cell Cardiol. (2017) 108:181–93. 10.1016/j.yjmcc.2017.06.006 28641979 PMC5548301

[111] Intapad S Ojeda NB Dasinger JH Alexander BT. Sex differences in the developmental origins of cardiovascular disease. Physiology. (2014) 29:122–32. 10.1152/physiol.00045.2013 24583768 PMC3949204

[112] Sanghavi M Rutherford JD. Cardiovascular physiology of pregnancy. Circulation. (2014) 130:1003–8. 10.1161/CIRCULATIONAHA.114.009029 25223771

[113] Morrow EH. The evolution of sex differences in disease. Biol Sex Differ. (2015) 6:1–7. 10.1186/s13293-015-0023-0 25774286 PMC4359385

[114] Cox RM Calsbeek R. Sexually antagonistic selection, sexual dimorphism, and the resolution of intralocus sexual conflict. Am Nat. (2009) 173:176–87. 10.1086/595841 19138156

[115] Pennell TM Morrow EH. Two sexes, one genome: the evolutionary dynamics of intralocus sexual conflict. Ecol Evolution. (2013) 3:1819–34. 10.1002/ece3.540 23789088 PMC3686212

[116] Hosken DJ Archer CR Mank JE. Sexual conflict. Curr Biol. (2019) 29:R451–5. 10.1016/j.cub.2019.03.052 31163156

[117] Parsch J Ellegren H. The evolutionary causes and consequences of sex-biased gene expression. Nat Rev Genet. (2013) 14:83–7. 10.1038/nrg3376 23329110

[118] Rowe L Chenoweth SF Agrawal AF. The genomics of sexual conflict. Am Nat. (2018) 192:274–86. 10.1086/698198 30016158

[119] Ingleby FC Flis I Morrow EH. Sex-biased gene expression and sexual conflict throughout development. Cold Spring Harbor Perspect Biol. (2015) 7:a017632. 10.1101/cshperspect.a017632 25376837 PMC4292171

[120] Gluckman PD Hanson MA Buklijas T. A conceptual framework for the developmental origins of health and disease. J Dev Origins Health Dis. (2010) 1:6–18. 10.1017/S2040174409990171 25142928

[121] Lea AJ Tung J Archie EA Alberts SC. Developmental plasticity: bridging research in evolution and human health. Evol Med Public Health. (2017) 2017:162–75. 10.1093/emph/eox019 29424834 PMC5798083

[122] Gluckman PD Hanson MA Low FM. Evolutionary and developmental mismatches are consequences of adaptive developmental plasticity in humans and have implications for later disease risk. Philos Trans R Soc Lond B Biol Sci. (2019) 374:20180109. 10.1098/rstb.2018.0109 30966891 PMC6460082

[123] Gabory A Attig L Junien C. Sexual dimorphism in environmental epigenetic programming. Mol Cell Endocrinol. (2009) 304:8–18. 10.1016/j.mce.2009.02.015 19433243

[124] Wizeman TM Pardue ML. Exploring the Biological Contributions to Human Health: Does Sex Matter? Washington, DC: National Academy Press (2001). 25057540

[125] Iorga A Cunningham CM Moazeni S Ruffenach G. Umar S, Eghbali M. The protective role of estrogen and estrogen receptors in cardiovascular disease and the controversial use of estrogen therapy. Biol Sex Differ. (2017) 8:33. 10.1186/s13293-017-0152-8 29065927 PMC5655818

[126] Ventura-Clapier R Moulin M Piquereau J Lemaire C Mericskay M Veksler V. Mitochondria: a central target for sex differences in pathologies. Clin Sci. (2017) 131:803–22. 10.1042/CS20160485 28424375

[127] Blekhman R Marioni JC Zumbo P Stephens M Gilad Y. Sex-specific and lineage-specific alternative splicing in primates. Genome Res. (2010) 20:180–9. 10.1101/gr.099226.109 20009012 PMC2813474

[128] Karp NA Mason J Beaudet al Benjamini Y Bower L Braun RE. Prevalence of sexual dimorphism in mammalian phenotypic traits. Nat Commun. (2017) 8:15475. 10.1038/ncomms1547 28650954 PMC5490203

[129] Shen JJ Wang TY Yang W. Regulatory and evolutionary signatures of sex-biased genes on both the X chromosome and the autosomes. Biol Sex Differ. (2017) 8:35. 10.1186/s13293-017-0156-4 29096703 PMC5668987

